# Supplementary material for: Phase I/II Clinical Trial of the Anti-Podoplanin Monoclonal Antibody Therapy in Dogs with Malignant Melanoma
Source: Cells. 2020 Nov 23;9(11):2529. doi: 10.3390/cells9112529 (PMC7700559; doi:10.3390/cells9112529)
Supplement: Supplementary file 1 [file cells-09-02529-s001.zip › Cells supple fig/Supple_Legend.docx]

**Supplemental Figure Legends**

**Supplemental Figure 1.** Flow cytometric analysis on CHO, CHO/dPDPN, KMeC, CMM1, CMM2, CMM11 and CMM8 cells which were treated with isotype control (black line) and P38Bf (red line). P38Bf showed higher fluorescence intensity against CHO/dPDPN, CMM1 and CMM11 cells compared with CHO, KMeC, CMM2 and CMM8 cells.

**Supplemental Figure 2.** Safety trial of P38Bf in a healthy beagle. Kinetics of P38Bf in blood serum. P38Bf reached a peak at 1 hour after administration, and gradually decreased for 2 weeks. The vertical dashed line shows the analytical limit of detection (3.9 μg/mL).

**Supplemental Figure 3.** Evaluation of acute adverse effect of P38Bf. The dog was monitored up to 5 hours after intravenous administration of P38Bf. Body temperature, heart rate, respiratory rate and percutaneous oxygen saturation (SpO2) were recorded at baseline and every 30 minutes post P38Bf administration. Through the monitoring, no significant changes were observed.

**Supplemental Figure 4.** Evaluation of chronic adverse effect of P38Bf. General physical examination, CBC, serum chemistry, glomerular filtration rate test, was performed for 8 weeks after administration. No significant changes were observed. The vertical dashed line shows reference value.

**Supplemental Figure 5.** Histopathological analysis of the dog administrated P38Bf. Abnormal findings were not observed in histopathological examination including PDPN positive normal tissues: lung, kidney and lymphatic vessel of duodenum. Scale bar of lung, kidney and others, 100 μm, 100 μm and 500 μm, respectively.

**Supplemental Figure 6.** Representative images of immunohistochemistry for dPDPN in oral malignant melanomas of three cases. Tissues of Dog#1 and Dog#2 were stained with alkaline phosphatase and the tissue of Dog#3 was stained with a horseradish peroxidase. Enlarged images are shown in black square.

**Supplemental Figure 7.** Serum CRP levels of Dog#1, Dog#2 and Dog#3 before and after administration of P38Bf. CRP was increased in all dogs after administration of P38Bf. Black arrows indicate the timing of P38Bf administration.

**Supplemental Figure 8.** Radiographic evaluation of lung metastasis of Dog#1. The lung metastatic lesions tended to develop.

**Supplemental Figure 9.** Computed tomographic evaluation of the primary tumor at oral cavity of Dog#2. Primary lesion tended to develop.
