# Supplementary figures and images for: Phase I/II Clinical Trial of the Anti-Podoplanin Monoclonal Antibody Therapy in Dogs with Malignant Melanoma
Source: Cells. 2020 Nov 23;9(11):2529. doi: 10.3390/cells9112529 (PMC7700559; doi:10.3390/cells9112529)

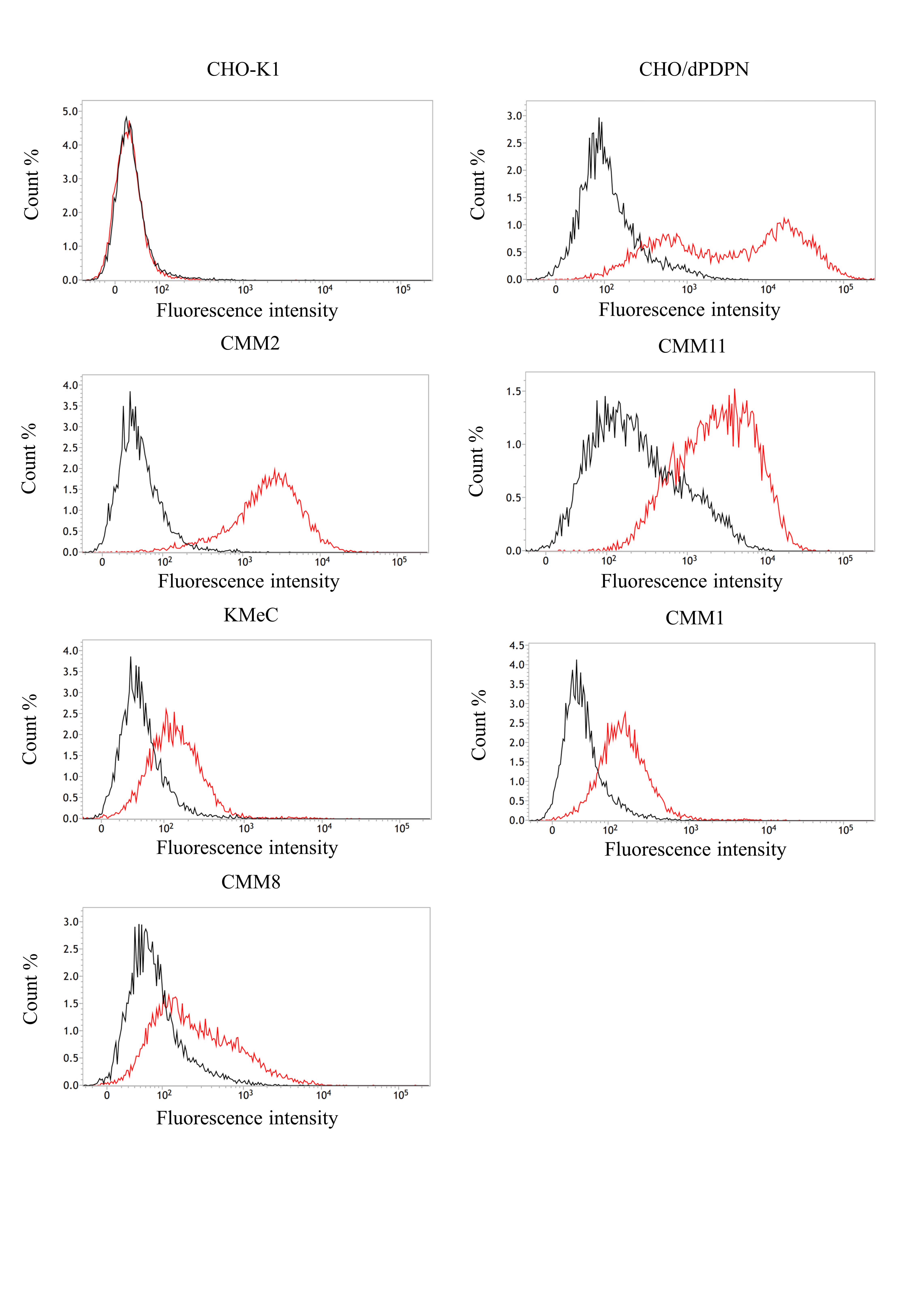

Supplement: Supplementary file 1 [file cells-09-02529-s001.zip › Cells supple fig/Fig S1.tif]

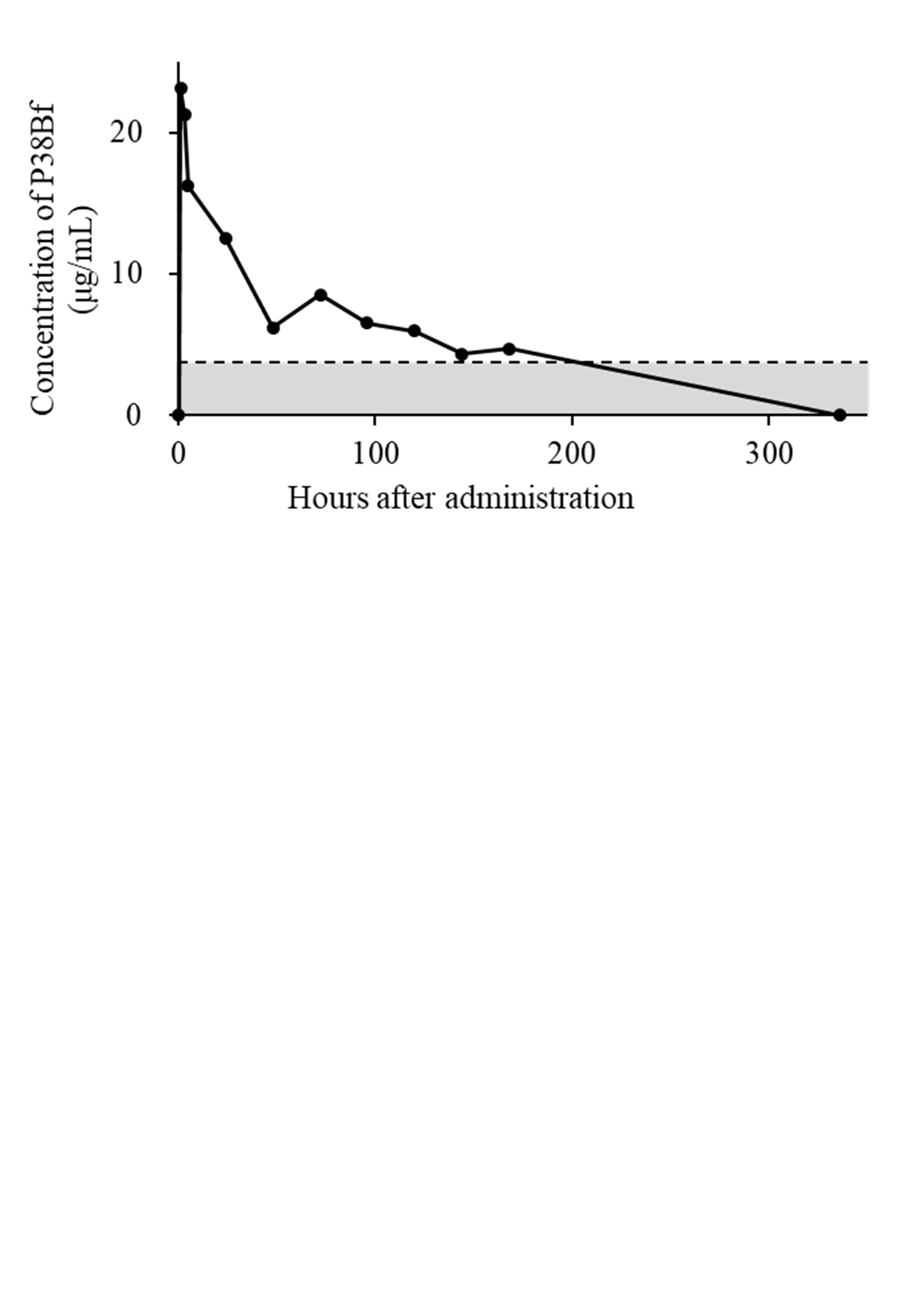

Supplement: Supplementary file 1 [file cells-09-02529-s001.zip › Cells supple fig/Fig S2.tif]

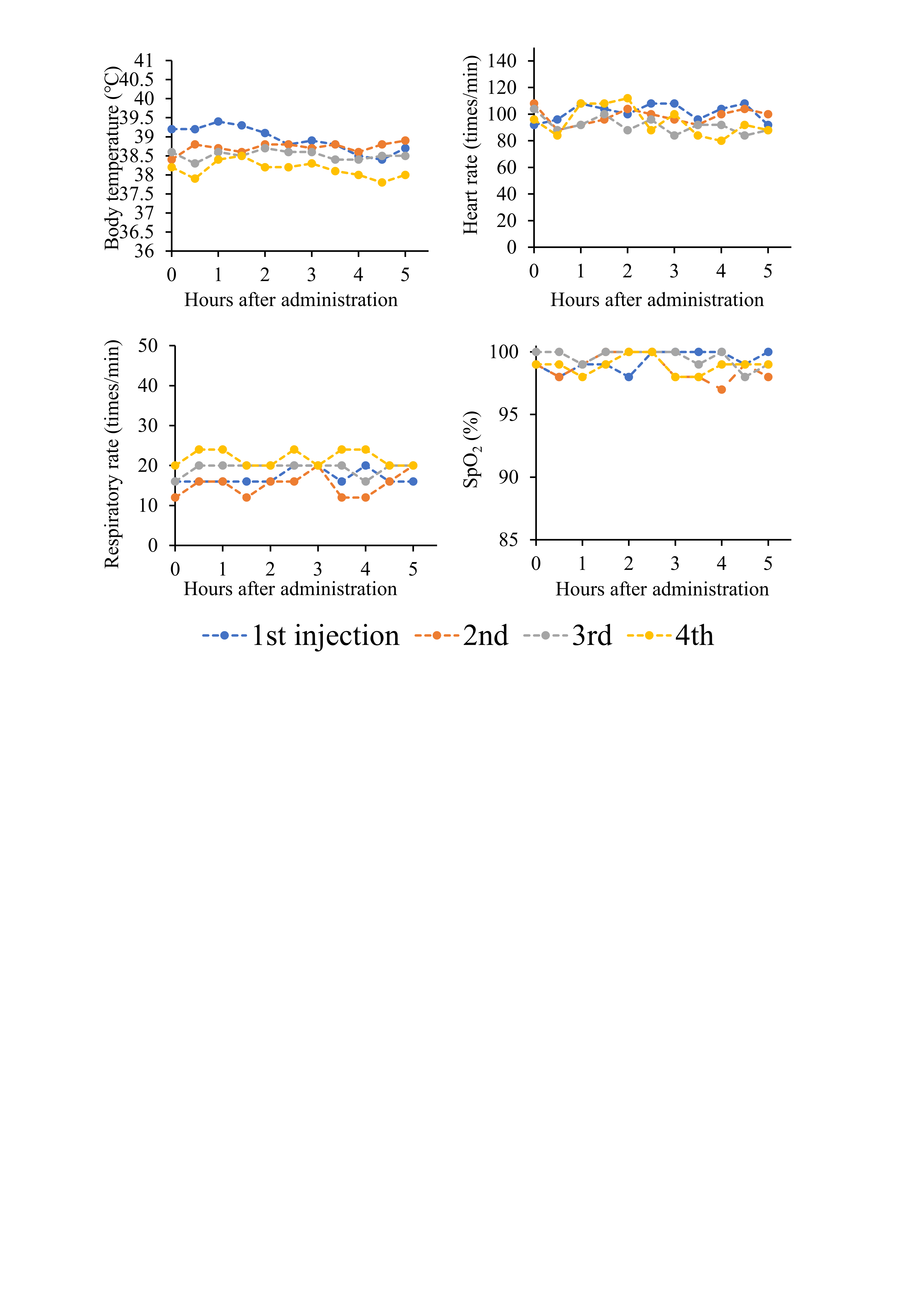

Supplement: Supplementary file 1 [file cells-09-02529-s001.zip › Cells supple fig/Fig S3.tif]

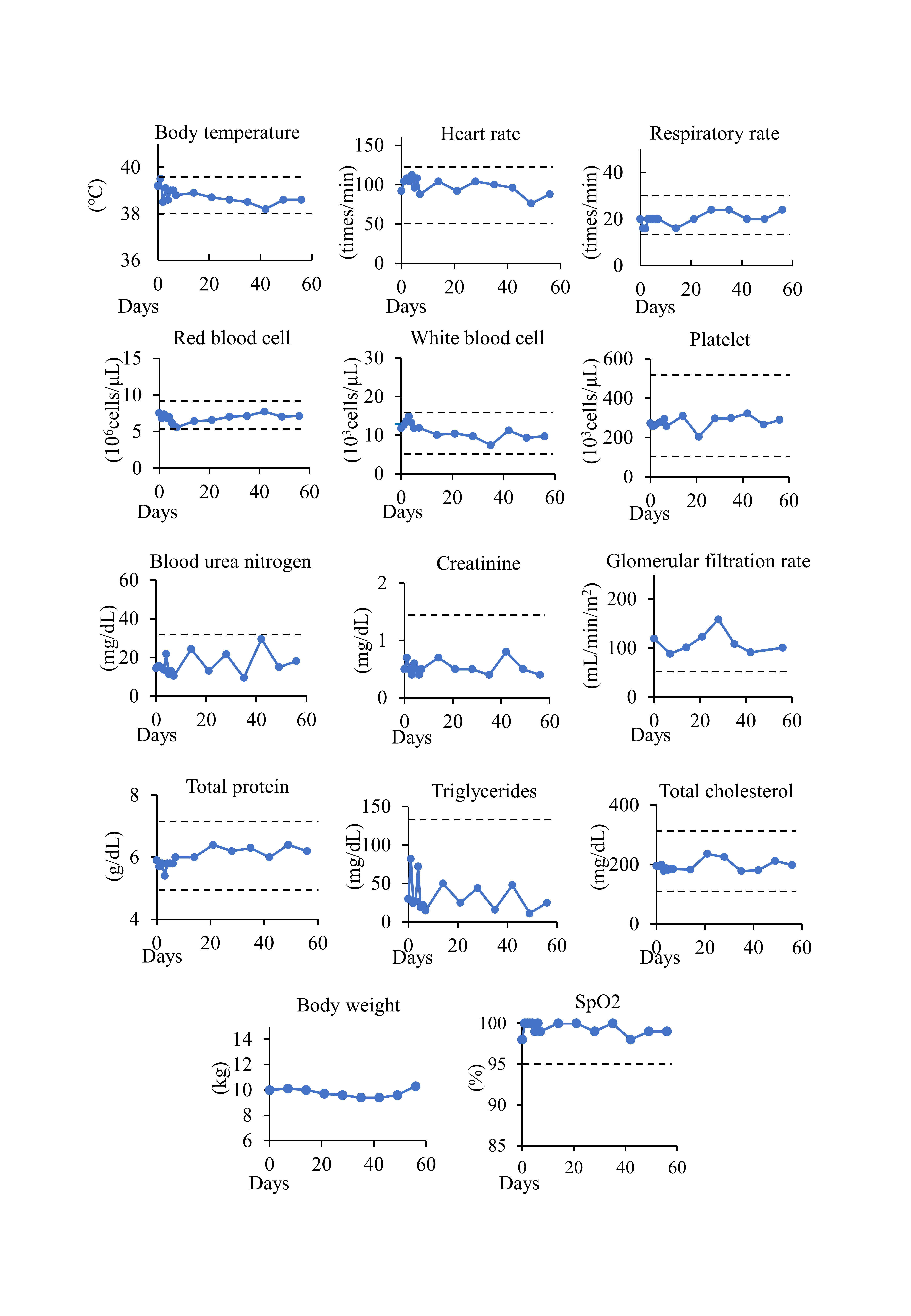

Supplement: Supplementary file 1 [file cells-09-02529-s001.zip › Cells supple fig/Fig S4.tif]

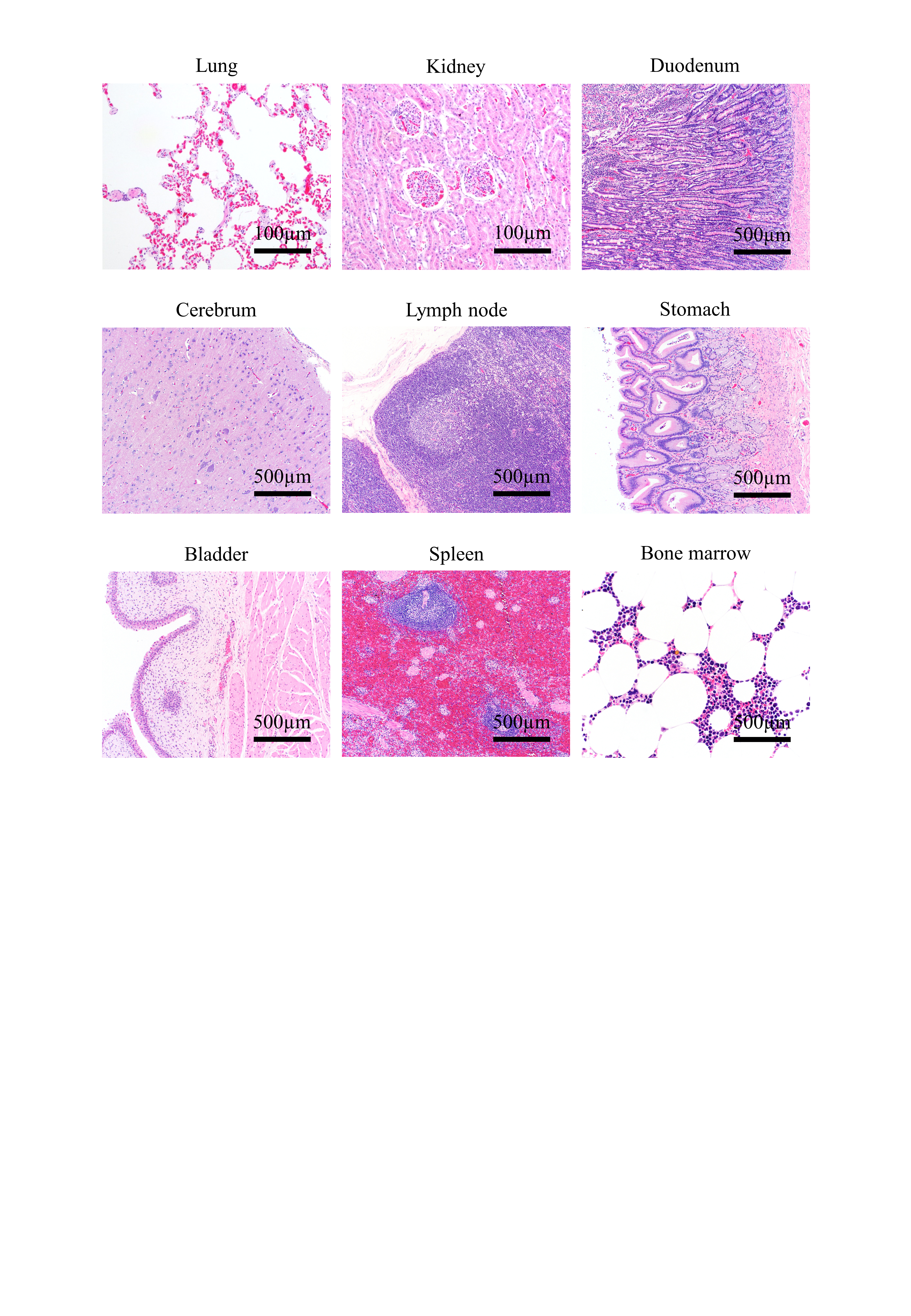

Supplement: Supplementary file 1 [file cells-09-02529-s001.zip › Cells supple fig/Fig S5.tif]

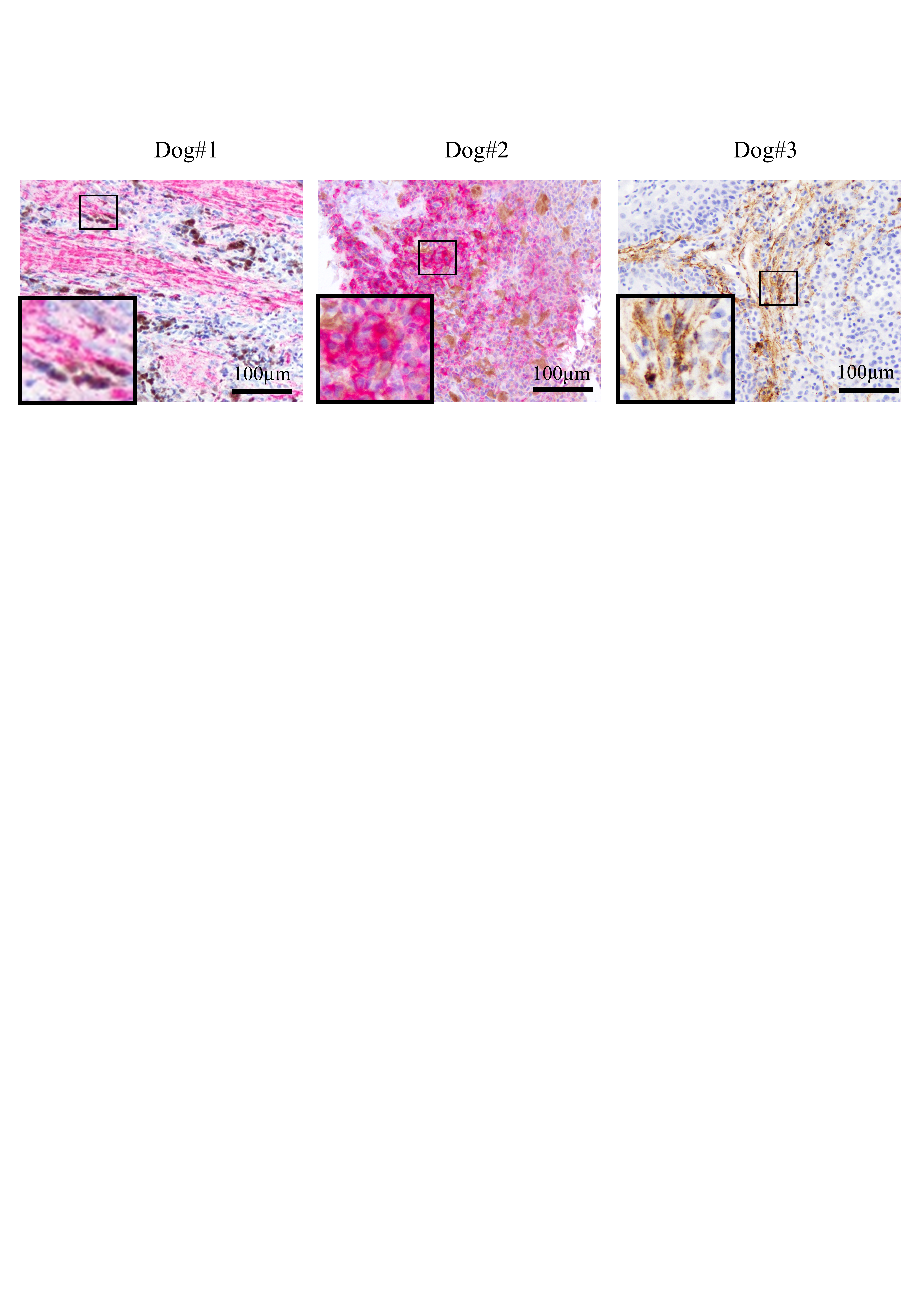

Supplement: Supplementary file 1 [file cells-09-02529-s001.zip › Cells supple fig/Fig S6.tif]

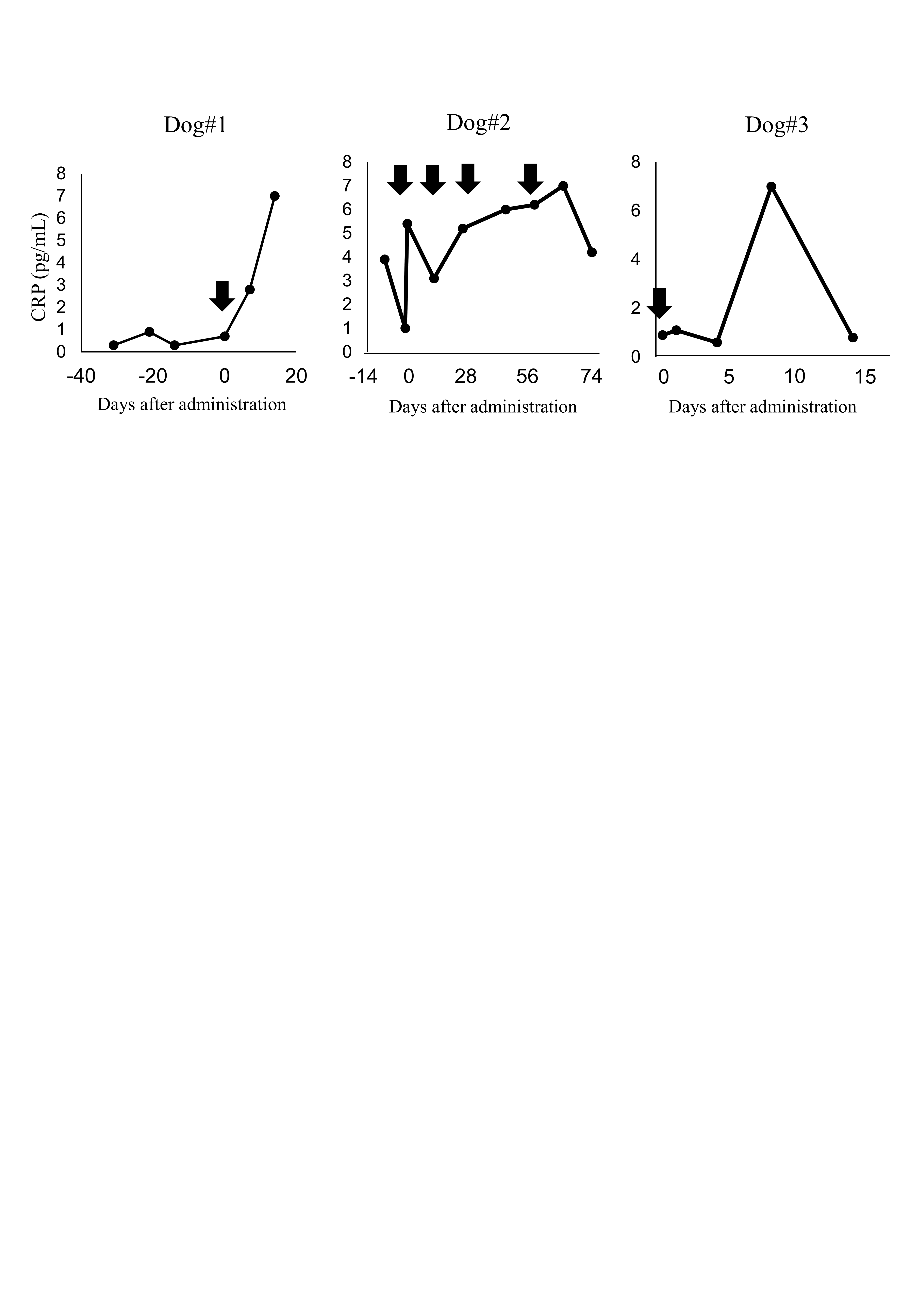

Supplement: Supplementary file 1 [file cells-09-02529-s001.zip › Cells supple fig/Fig S7.tif]

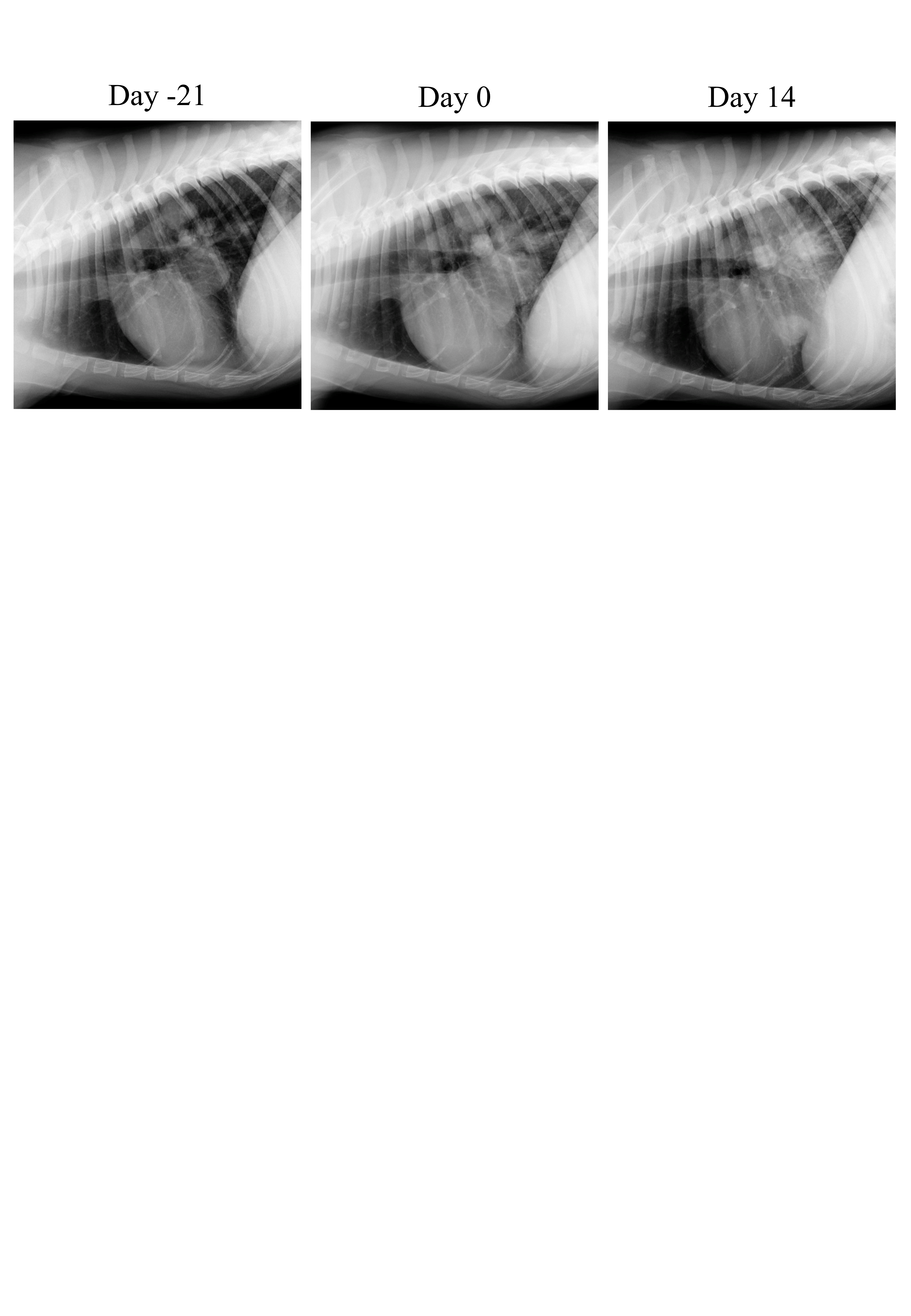

Supplement: Supplementary file 1 [file cells-09-02529-s001.zip › Cells supple fig/Fig S8.tif]

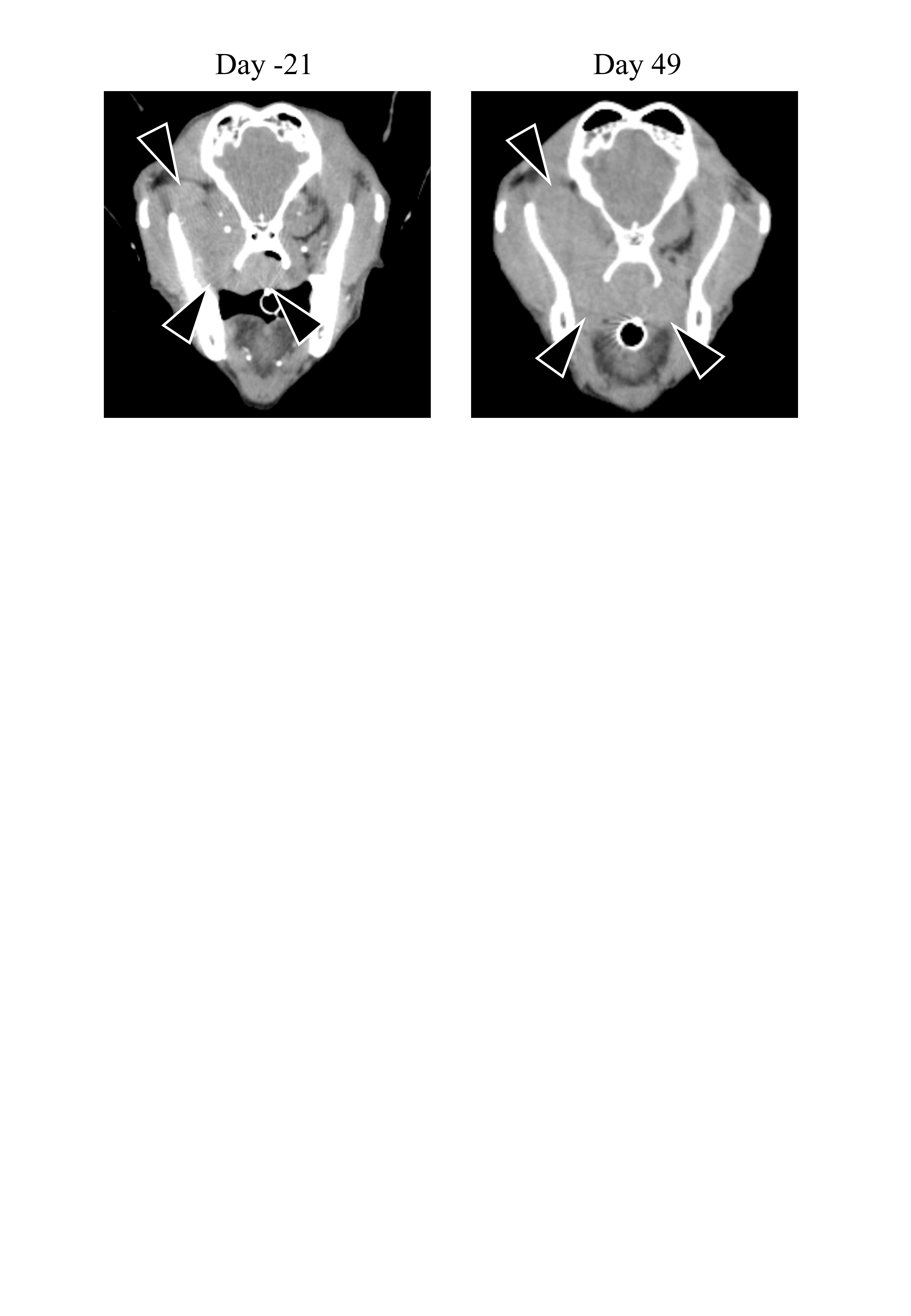

Supplement: Supplementary file 1 [file cells-09-02529-s001.zip › Cells supple fig/Fig S9.tif]

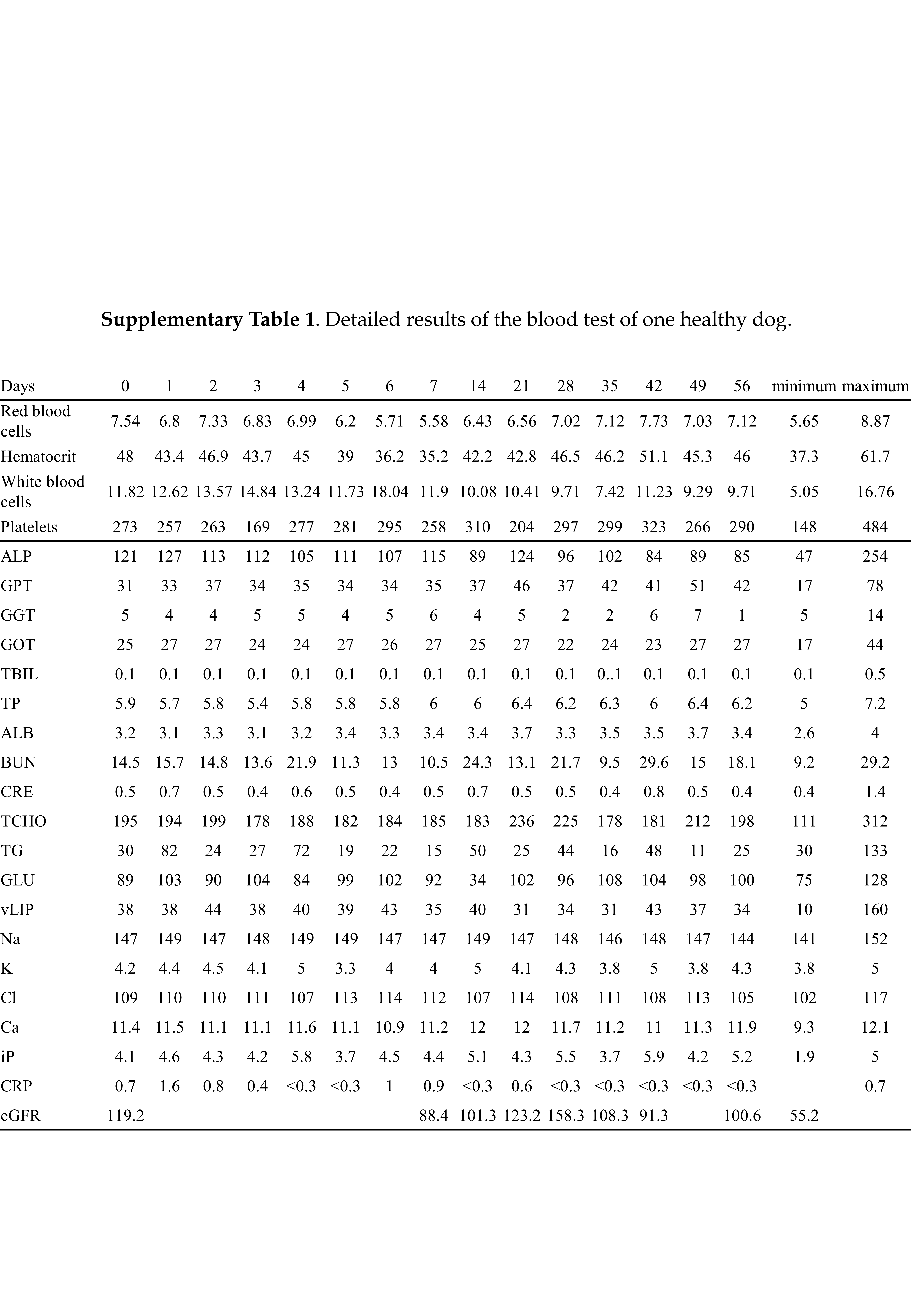

Supplement: Supplementary file 1 [file cells-09-02529-s001.zip › Cells supple fig/Table S1.tif]
